# Crimean-Congo Hemorrhagic Fever among Health Care Workers, Turkey

**DOI:** 10.3201/eid2003.131353

**Published:** 2014-03

**Authors:** Aysel Kocagul Celikbas, Başak Dokuzoğuz, Nurcam Baykam, Sebnem Eren Gok, Mustafa Necati Eroğlu, Kenan Midilli, Herve Zeller, Onder Ergonul

**Affiliations:** Ankara Numune Training and Research Hospital, Ankara, Turkey (A.K. Celikbas, B. Dokuzoğuz, N. Baykam, S.E. Gok, M.N. Eroğlu);; Istanbul University, Istanbul, Turkey (K. Midilli);; Institut Pasteur, Lyon, France (H. Zeller);; Koç University, Istanbul (O. Ergonul)

**Keywords:** Crimean-Congo hemorrhagic fever, Turkey, viruses, health care workers, Bunyaviridae, vector-borne infections, zoonoses

## Abstract

We investigated 9 cases of Crimean-Congo hemorrhagic fever (1 fatal, 2 asymptomatic) among health care workers in Turkey. Needlestick injuries were reported for 4 workers. Eight received ribavirin. In addition to standard precautions, airborne infection isolation precautions are essential during aerosol-generating procedures. For postexposure prophylaxis and therapy, ribavirin should be given.

Crimean-Congo hemorrhagic fever (CCHF) has been described from Africa, Asia, southeastern Europe, and the Middle East (*1*). The CCHF virus (CCHFV) belongs to the family *Bunyaviridae*, genus *Nairovirus*, and causes severe disease in humans; the reported case-fatality rate is 3%–30% (*1*). Humans become infected through the bites of ticks, contact with infected patients' body fluids, or contact with blood or tissues from viremic livestock. Health care workers (HCWs) are at occupational risk for CCHFV infection. Health care–related CCHFV infections have been reported in Pakistan (*2*–*5*), the United Arab Emirates (*6*), South Africa (*7*), Iran (*8*), India (*9*), Tajikistan (*10*), and Turkey (*11*). We describe the outcomes of 9 HCWs in Turkey who had occupational exposure to CCHFV.

## The Cases

The 9 HCWs and all CCHF patients under their care were admitted to the Infectious Diseases and Clinical Microbiology clinic (IDCM) of Ankara Numune Education and Research Hospital (Ankara, Turkey) during 2004–2011 with confirmed CCHF. All 9 HCWs were aware of possible or confirmed CCHFV infection in their patients. During this period, ≈7,000 confirmed CCHF cases were recorded in Turkey; nearly 300 of these patients were hospitalized in IDCM. Acute- and convalescent-phase serum samples from the index patients were sent to the national reference laboratory of Turkey. CCHFV infection was confirmed through IgM positivity by ELISA and/or positive PCR results for CCHFV in blood. After episode 1, the HCWs’ serum samples were sent to the Pasteur Institute (Lyon, France) for contact tracing. The HCWs’ infections were scored according to a severity score index (*12*). In episode 3, to investigate the source of infection, molecular techniques were used. Oral ribavirin for treatment was administered at the dosage recommended by the World Health Organization (4 g/d for 4 d, 2.4 g/d for 6 d), and for prophylaxis (2g/4×/d for 7 d). The index patients and the HCWs were given erythrocyte, fresh frozen plasma, and total blood preparations depending on their homeostasis.

### Episode 1

In 2005, CCHFV infection was diagnosed in a woman on the day of delivery by cesarean section. She was transferred to IDCM, and her baby was transferred to the newborn service at the Dr. Sami Ulus Children's Hospital (Ankara, Turkey). A nurse in IDCM, who had fever and myalgia, was later found to have CCHFV infection (Table 1). Transmission was related to the improper use of gloves during the care of the mother’s surgical wound. The mother recovered, but the infant died 5 days after birth. A nurse in the neonatal clinic in the children’s hospital also acquired CCHFV infection, which was attributed to the intubation and aspiration of bloody secretions from the baby without proper use of gloves and mask. For both HCWs, the incubation period was 2 days. The first HCW was given ribavirin at symptom onset. The second nurse’s illness was mild; because she had a potential for getting pregnant, ribavirin was not started. Both nurses recovered completely (Table 2).

**Table 1:** Clinical and laboratory findings of HCWs in whom Crimean-Congo hemorrhagic fever developed after occupational exposure, Turkey, 2004–2011*†

HCW, outcome	Body temperature, °C	Bleeding	Leukocytes/mm^3^	Platelets/mm^3^	AST	ALT	APTT	Fibrinogen	SSI
1, survived	38.5	No	800	42,000	425	346	44	225	Moderate
2, survived	37.2	No	1100	53,000	145	81	43	270	Mild
3, died	40.5	Ecchymosis, hematemesis, melena, hematuria	11,100	40,000	251	277	90	171	Severe
4, survived	40.5	No	2,900	78,000	150	110	37.4	250	Mild
5, survived	39	Epistaxis	1,800	58,000	167	129	64	218	Moderate
6, survived	40.5	No	1,800	44,000	123	216	40.5	165	Moderate
7, survived	39.1	No	3,100	13,000	418	132	40.9	170	Moderate

*HCW, health care worker; AST, aspartate aminotransferase; ALT, alanine aminotransferase; APTT, activated partial thromboplastin time ; SSI, severity score index. †Reference values: leukocytes, 4,000–11,000/mm^3^; platelets, 150,000–450,000/mm^3^; AST, <50 IU/L; ALT, <50 IU/L; APTT, 24–36 sec; fibrinogen, 200–400 mg/dL.

**Table 2:** Demographic features of HCWs with occupational exposure to Crimean-Conger hemorrhagic fever virus, Turkey, 2004–2011*

Episode, outcome†	HCW age, y/sex/profession	Procedure	Transmission route	Ribavirin for postexposure prophylaxis	Ribavirin for therapy (no. d after symptom onset)	Fatal
Episode 1; survived, her baby died	36/M/nurse	Wound care	Contact with surgical wound without protective equipment	No	Yes (0)	No
	31/F/nurse	Intubation, aspiration	Aerosol and droplet and contact without protective equipment	No	No	No
Episode 2; died	28/F/nurse	Phlebotomy	Needlestick	No	Yes (3)	Yes
Episode 3; died	41/M/physician	Resuscitation	Aerosol and droplet	–	Yes (0)	No
	26/M/physician	Nasal tamponade	Indirect contact	–	Yes (0)	No
	29/M/physician	Nasal tamponade	Indirect contact	–	Yes (0)	No
Episode 4; survived	30/M/nurse	Phlebotomy	Needlestick	No	Yes (1)	No
Episode 5; survived	30/F/nurse	Phlebotomy	Needlestick	Yes	–	No
Episode 6; survived	24/F/physician	Phlebotomy	Needlestick	Yes	–	No

*HCW, health care worker; –, ribavirin not necessary. †Outcome for the index case-patient in each episode.

After this episode, the index patient’s contacts were traced. Serum samples from 37 HCWs at IDCM and the obstetrics and newborn clinics at the children’s hospital who were at risk for infection were investigated for CCHF. In addition to the 2 nurses, 2 nurses from the neonatal clinic were CCHF IgM positive but were asymptomatic.

### Episode 2

In 2006, a nurse received a needlestick injury during a phlebotomy of a CCHFV-infected patient. She was using gloves but no gown, and the needle stuck to her forearm. The infected patient died. The nurse’s symptoms began 2 days after the incident, and she was transferred to IDCM 4 days after the incident. At admission, her severity score index was high. She had ecchymosis, epistaxis, hematemesis, melena, vaginal bleeding, and somnolence (Table 1). She received oral ribavirin 5 days after the incident, which possibly had limited effect because her illness already had progressed to confusion and gastrointestinal bleeding. She died on the second day after hospital admission.

### Episode 3

In 2008, a patient with CCHFV infection and a high viral load (10^8^ copies/mL) was hospitalized with hemoptysis, hematemesis, melena, epistaxis, and intraalveolar bleeding. He died 3 days after admission. One infectious diseases resident and 2 otorhinolaryngology residents acquired CCHFV infection. All HCWs had worn personal protective equipment during the intervention. One otorhinolaryngology resident performed nasal tamponade. After the intervention, the other otorhinolaryngology resident handled and cleaned a head mirror without using gloves, although he was not in face-to-face contact with the patient. The infectious diseases resident resuscitated the patient without apparent direct contact with the patient’s bloody secretions. For the 3 residents, fever, malaise, and myalgia developed 2–5 days after exposure. Ribavirin was started at symptom onset. No contact was observed between the patient and the HCWs, but the RNA sequences from the patients and HCWs were identical by molecular techniques. All of the HCWs recovered.

The transmission of CCHFV could have resulted from indirect contact with contaminated devices, such as the head mirror; the improper removal of gowns, masks, gloves; inadequate hand hygiene; or failure to use N95 masks during aerosolizing procedures. During the procedures that could generate aerosols, HCWs should wear an N95 mask or FFP2 respirator (*13*). The patients with higher viral load were reported to be more severe disease (*14*).

### Episode 4

In 2008, a phlebotomist working in a children’s hospital had a needlestick injury during phlebotomy of a CCHFV-infected child. He was hospitalized 1 day after symptom onset, and ribavirin was started. His severity score index was moderate, and he recovered.

### Episodes 5 and 6

A nurse in 2007 and a pediatric resident in 2008 incurred needlestick injuries during phlebotomy of a CCHFV-infected patient. Postexposure prophylaxis with ribavirin was started immediately after the injuries, and no infections developed.

## Conclusions

Six of the 9 CCHF-infected HCWs reported here had histories of needlestick injuries or contact with contaminated blood without adequate barrier precautions. An integrated strategy for controlling accidental exposure to body fluids was developed to protect HCWs against CCHFV infection. All personnel, including cleaning staff in health care units on all shifts were informed and trained about the transmission risks, protection, and clinical symptoms of CCHF (*13*). The standard, contact, and droplet precautions were usually sufficient to protect against CCHFV infection during the routine care of CCHF patients. In addition to the practices of previous years, the airborne infection isolation precautions during aerosol-generating procedures were performed. The number of HCWs caring for patients with severe CCHF was limited. After all of these measures were enforced, occupational CCHFV infection did not occur in IDCM.

Ribavirin is an effective treatment for CCHFV infection (*12*) and beneficial for postexposure prophylaxis (*13*,*15*). Therapy should be started as early as possible. In the 2 HCWs reported here who received ribavirin for postexposure prophylaxis, no symptoms developed; similar reports will be useful for increasing the power of this conclusion. We used a ribavirin dosage of 2g/day, but no consensus exists about the dosage for postexposure prophylaxis.
